# Non-Islet Cell Tumor Hypoglycemia Secondary to a 20 cm Intra-Abdominal Leiomyoma in a Male Patient: A Case Report and Literature Review

**DOI:** 10.1155/2024/6651107

**Published:** 2024-06-14

**Authors:** Michelle D. Lippincott, James D. McDonald, Marilyn M. Bui, Ricardo J. Gonzalez, Rachel K. Voss

**Affiliations:** ^1^ Department of Surgery University of South Florida, Tampa, FL, USA; ^2^ Department of Sarcoma Oncology H. Lee Moffitt Cancer Center and Research Institute Tampa, Tampa, FL, USA; ^3^ Department of Pathology H. Lee Moffitt Cancer Center, Tampa, FL, USA

## Abstract

Non-islet cell tumor hypoglycemia (NICTH) is a rare clinical entity associated with large mesenchymal tumors. Its pathogenesis is most commonly mediated by tumor overproduction of “big” insulin-like growth factor-2. Here, we present a 54-year-old male who presented with noninsulin-mediated hypoglycemia and a 20 cm intra-abdominal leiomyoma. His hypoglycemic episodes resolved after the resection of his tumor. To our knowledge, this is the only documented case in the English literature of NICTH associated with leiomyoma in a male patient. NICTH due to a benign leiomyoma should be in the differential diagnosis for any patient with hypoglycemia and an abdominal mass.

## 1. Introduction

Non-islet cell tumor hypoglycemia (NICTH) is a syndrome defined as fasting hypoglycemia caused by noninsulin-producing tumors. The true incidence of NICTH is unknown but is thought to be less common than its insulinoma counterpart [1]. The pathophysiology of hypoglycemia in these tumors is secondary to the secretion of high molecular weight insulin-like growth factor-2 (IGF-2), also termed “big” IGF-2 in the literature [2]. This protein variant mimics the effects of insulin in vivo [3]. Here, we present a case of benign intra-abdominal leiomyoma that presented with refractory hypoglycemia, which we suspect was due to tumor secretion of IGF-2 leading to NICTH. To our knowledge, this is the first reported case of NICTH associated with leiomyoma in a male patient.

## 2. Case Report

### 2.1. Presentation

Our patient is a 54-year-old male with no relevant medical history including no history of diabetes. He initially presented to an outside hospital after being found at home unconscious with a blood glucose of 18 mg/dL. He was treated with oral glucose by emergency medical services and promptly returned to his baseline normal mental status. In retrospect, he reported approximately 2 months of neuroglycopenic symptoms such as weakness, fatigue, and brain fog that resolved upon eating but no prior episodes of losing consciousness. He was brought to a local emergency room for further evaluation. On examination, he was noted to have a large palpable, painless abdominal mass. Further history revealed a significant drinking history with a reported alcohol intake of six drinks per day on weekdays and 12 drinks per day on weekends.

### 2.2. Workup

**Figure 1:**
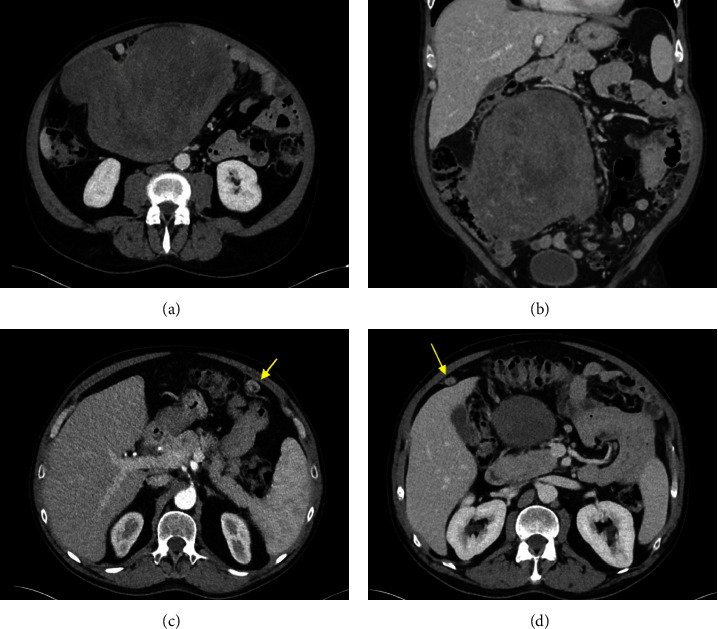
Representative images of the patient's CT abdomen/pelvis with IV contrast showing a large, lobulated heterogeneous abdominal mass in images (a) and (b) and omental nodules in images (c) and (d).

The patient was admitted for further workup to the outside hospital. His laboratory evaluation was notable for low insulin, low proinsulin, and normal beta-hydroxybutyrate levels, as listed in Table 1. His liver function panel, renal function, and complete blood count were normal (Table 1). He underwent computed tomography (CT) scan of the abdomen and pelvis, which showed a lobulated, heterogeneous abdominal mass measuring 20.5 × 18 × 16.5 cm with mesenteric lymphadenopathy and omental nodules concerning for metastatic disease (Figure 1). There was an associated mass effect on the colon and inferior vena cava. Ultrasound-guided core needle biopsy was performed and showed benign-appearing smooth muscle suspicious for leiomyoma.

### 2.3. Treatment and Outcomes

Throughout his hospital stay, he demonstrated refractory fasting hypoglycemia ranging from 50 to 69 mg/dL requiring the administration of 50% dextrose solution (D50), which would successfully resolve his hypoglycemia for a few hours. He was ultimately discharged home with outpatient endocrinology follow-up. He was started on an oral dexamethasone taper by his outside endocrinologist, which decreased the number of hypoglycemic episodes to a few per day. He was prescribed a continuous glucose monitor to alert him to hypoglycemia. He noted that he had to ingest food every few hours to prevent ongoing hypoglycemic episodes. His endocrinologist ultimately referred him to our tertiary care center for surgical resection of the abdominal mass.

Three months after the start of his symptoms, he presented to our sarcoma clinic. His body mass index (BMI) was 28.9, and he had mild hypertension (150/95 mmHg), but all other vital signs were normal at the initial consultation visit. His pathology was reviewed by one of our center's sarcoma pathologists, who agreed the biopsy was consistent with benign smooth muscle. Due to the concern about his life-threatening recurrent hypoglycemia, we recommended expeditious excision of the mass. Our suspicion was high for a paraneoplastic syndrome and possible undiagnosed malignancy. At the time of presentation, we suspected he had Doege–Potter syndrome due to a solitary fibrous tumor (SFT) and sampling error of his ultrasound-guided biopsy. He was not on glucocorticoids at the time of his resection.

He was promptly taken to the operating room 3 days after his initial clinic visit where he underwent an exploratory laparotomy. Findings were notable for 20 × 10 × 14 cm mass originating from the transverse colon mesentery. It had formed attachments to the surrounding small bowel mesentery and colonic epiploica with notable neovascularization. The tumor was excised along with approximately 15 cm of the central transverse colon and its mesentery, and a primary colocolonic anastomosis was performed. Several omental nodules 1–2 cm in size were palpated (as seen on imaging) so a complete greater omentectomy was performed. One of these omental nodules was sent for frozen analysis and showed benign lymphoid tissue. There was also palpable adenopathy in the small bowel and right colon mesentery, but it was soft in character. One firmer small bowel mesenteric nodule was encountered and was excised. Estimated blood loss was 200 mL, and the total surgical time was 3 hr and 43 min. He ultimately tolerated the procedure well and was admitted postoperatively for recovery and blood glucose monitoring.

Initially, he had glucose checks every 4 hr, then every 6 hr. He had no episodes of hypoglycemia postoperatively. His glucose levels ranged from 84 to 178 mg/dL (Table 1), and he did not require any extra dextrose supplementation. He had return of bowel function on postoperative day 3, and he was discharged home on postoperative day 5. On postoperative follow-up visit to our sarcoma clinic 2.5 weeks after surgery, he reported no further episodes of hypoglycemia and was recovering well with no apparent complications.

## 3. Histology

**Figure 2:**
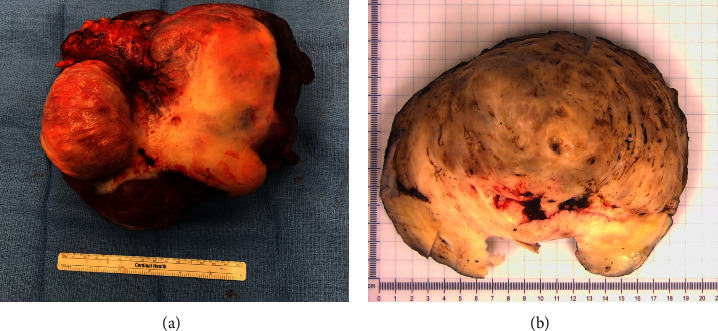
Images of the resected specimen. (a) Photo of the specimen after resection. The section of resected colon is in the upper left corner of the photo. (b) Photo of cross-section of the leiomyoma after formalin fixation.

**Figure 3:**
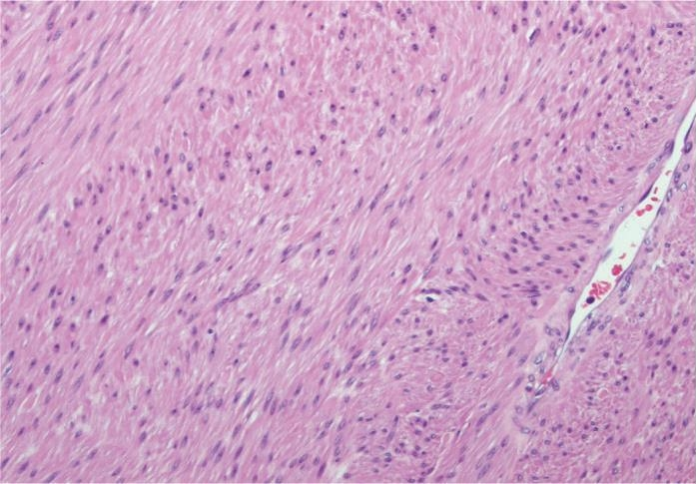
A 200x magnification microscopic image of the tumor with hematoxylin and eosin staining showing tumor cells with bland spindle-shaped nuclei and eosinophil cytoplasm arranged in intersecting fascicles. A large thin-wall benign vessel is noted at the right side of the image.

Final pathology revealed a benign leiomyoma measuring 20.5 × 18 × 13 cm. Its gross appearance was pink-tan and encapsulated with adherence to the colonic serosa (Figure 2). The tumor was mostly solid and composed of bland leiomyoma cells with degenerative changes of ischemic necrosis, hyalinization, and myxoid change (Figure 3). The colon was uninvolved and surgical margins were negative. The small bowel mesenteric nodule was a benign fibrotic nodule. The omentum contained benign fibrotic and chronically inflamed tissue with 10 benign lymph nodes and no evidence of additional leiomyomas. Additional molecular testing on the tumor using our institutional, next-generation sequencing MoffittSTAR panel revealed no significant genetic findings or mutations.

## 4. Discussion

NICTH is a rare paraneoplastic syndrome classified as recurrent hypoglycemia associated with noninsulin-producing tumors [4, 5, 6]. The most common mechanism of hypoglycemia is the production of high molecular weight IGF-2 [7, 8, 9]. Historically, NICTH has been reported to be associated with tumors of mesenchymal and epithelial origin. However, they have been associated with a wide variety of nonmesenchymal tumors, including hepatomas, gastric cancers, and colon cancer, to name a few [2]. Although it has been described in dogs [7, 8, 9] and women with uterine fibroids [10], to our knowledge, this is the first described case of NICTH associated with leiomyoma in a male patient.

In a similar case report, Ndzengue et al. [10] described an 80-year-old woman with preexisting diabetes who presented with neuroglycopenic symptoms and hypoglycemia. Similar to our patient, she was noted to have an abdominal mass on physical examination with sonographic findings of fibromyoma. She was bridged to surgery with dextrose infusions and steroids. She ultimately underwent a curative hysterectomy. Her preoperative workup was consistent with the profile of NICTH, including decreased levels of IGF-1 and a ratio of IGF-2 : IGF-1 > 10 [10].

NICTH-associated mesenchymal tumors are usually large—greater than 10 cm—by the time hypoglycemia manifests [2, 4, 11, 12]. Approximately half of patients with this syndrome will present with hypoglycemia prior to the discovery of an underlying tumor [2]. Our patient's presentation was similar to these reports. Hypoglycemia was his presenting symptom, and his large, undiagnosed abdominal mass was only identified on further investigation. This emphasizes the importance of obtaining cross-sectional imaging in patients with hypoinsulinemic hypoglycemia as abdominal and thoracic tumors can be inappreciable on examination.

Lab testing in these patients is notable for low levels of insulin, proinsulin, C-peptide, and beta-hydroxybutyrate [2, 5, 6]. Some patients have been reported to present with hypokalemia though this was not the case with our patient [2, 13]. Our patient's lab findings were consistent with the described profile of NICTH in the current literature. Additionally, described laboratory evaluation includes reduced levels of IGF-1 and a ratio of IGF-2 : IGF-1 > 10 [12]. Unfortunately, these biochemical studies were not performed on our patient prior to resection, and IGF-2 staining was not available at our facility to perform immunohistochemical staining on the specimen. Therefore, the diagnosis of NICTH is suspected to be due to IGF-2 in this case but we were unable to objectively prove this. There is no known inherited syndrome associated with NICTH related to a mesenchymal mass, but this presents another area for future investigation. Our 170-gene MoffittSTAR panel did not encounter any significant genetic alterations that would point to an inherited syndrome.

One entity that may be most familiar to oncology specialists is Doege–Potter syndrome. This syndrome is a type of NICTH specifically associated with SFTs. SFTs are most commonly found in the pleural cavity but can occur throughout the body and have variable metastatic potential [14, 15]. As with other described NICTH tumors, the pathogenesis of hypoglycemia in Doege–Potter syndrome is thought to be secondary to tumor secretion of “big” IGF-2. Definitive treatment of DPS involves resection of the tumor and management of hypoglycemic episodes with steroids or glucose [14, 15, 16]. Although DPS is a relatively well-known cause of tumor-related hypoglycemia, our case highlights the need to maintain a broad oncologic differential when assessing a patient with suspected NICTH.

Our patient's symptoms were managed medically with glucocorticoids which remains one of the most effective described medical therapies for this syndrome. Glucocorticoids both stimulate gluconeogenesis and suppress high molecular weight IGF-2 [17]. Additional described therapies include recombinant human growth hormone and tumor-targeted therapies. In congruence with previously reported cases, resection of the tumor was curative in the case of our patient. Although the final histology was benign, given the extreme rarity of this case, follow-up CT imaging of his abdomen and pelvis every 6 months for a few years was recommended by our institution's tumor board. The patient remains without evidence of recurrence or hypoglycemic symptoms now 2.5 years after surgery.

## 5. Conclusion

In conclusion, this was a 54-year-old male who developed NICTH secondary to a large intra-abdominal leiomyoma. He was successfully treated with glucocorticoids as a bridge to definitive surgical resection. Removal of the tumor resolved his hypoglycemic episodes, and he remains without evidence of recurrence.

## Figures and Tables

**Table 1:** Laboratory values at initial presentation, before surgery, and after surgery.

Laboratory test	Outside facility	Preoperatively	Postoperatively
Glucose (mg/dL)	*50–69 (L)*	84	84–178
Insulin (uIU/mL)	*0.39 (L)*	—	—
Proinsulin (pmol/L)	*0.8 (L)*	—	—
Beta-hydroxybutyrate (mmol/L)	0.3	—	—
Albumin (g/dL)	3.9	4.1	3.7
AST (U/L)	19	12	10
ALT (U/L)	17	17	11
Bilirubin (mg/dL)	0.7	0.5	1.1
Alkaline phosphatase (U/L)	43	48	43
INR	1.2	1.1	*1.3 (H)*
Blood urea nitrogen (mg/dL)	*4 (L)*	9	10
Creatinine (mg/dL)	*0.43 (L)*	*0.5 (L)*	*0.6 (L)*
Serum cortisol (ug/dL)	5.7	—	—

Italic text denotes abnormal values. (L), low value; (H), high value; and —, not available/not done.
